# Palladium anchored to BisPyP@bilayer-SiO_2_@NMP organic–inorganic hybrid as an efficient and recoverable novel nanocatalyst in suzuki and stille C–C coupling reactions

**DOI:** 10.1038/s41598-024-59666-4

**Published:** 2024-04-18

**Authors:** Eid Ahmed Abdalrazaq, Hala Kh. Mohammed, Daria K. Voronkova, Sanjeev Kumar Joshi, Ebraheem Abdu Musad Saleh, Anaheed Hussein Kareem, Abhinav Kumar, Ahmed Alawadi, Ali Alslaami, Rohollah Fathollahi

**Affiliations:** 1 Department of Chemistry, Faculty of Science, Al Hussein Bin Talal University, Ma’an, Jordan; 2 Medical Laboratory Techniques Department, Almaarif University College, Ramadi, Iraq; 3 Department of Mathematics and Natural Sciences, Gulf University for Science and Technology, Mishref Campus, Mubarak Al-Abdullah, Kuwait; 4 Bauman Moscow State Technical University Moscow, Moscow, Russia; 5 Department of Mechanical Engineering, Uttaranchal Institute of Technology, Uttaranchal University, Dehradun, 248007 India; 6 Department of Chemistry, College of Arts and Science, Prince Sattam Bin Abdulaziz University, 11991 Wadi Al-Dawasir, Saudi Arabia; 7 College of Health and Medical Technology, Al-Ayen University, Thi-Qar, 64001 Iraq; 8 Department of Nuclear and Renewable Energy, Ural Federal University Named After the First President of Russia Boris Yeltsin, Ekaterinburg, Russia 620002; 9 College of Technical Engineering, The Islamic University, Najaf, Iraq; 10 College of Technical Engineering, The Islamic University of Al Diwaniyah, Al Diwaniyah, Iraq; 11 College of Technical Engineering, The Islamic University of Babylon, Babylon, Iraq; 12 College of Medical Technique, The Islamic University, Najaf, Iraq; 13 Takin Shimi Sepanta Industries Co, Sirvan Industrial Zone, PO 6958140120, Ilam, Iran

**Keywords:** Cross-coupling reactions, Aryl halide, Phenylboronicacid, Triphenyltin chloride, Magnetic/bilayer silica mesostructures, Pd-BisPyP@Bilayer-SiO_2_@NMP, Chemistry, Nanoscience and technology

## Abstract

The palladium anchored to BisPyP@bilayer-SiO_2_@NMP organic–inorganic hybrid was employed as an effective and recyclable organometallic catalyst in Suzuki and Stille C–C coupling reactions. The structure of this magnetic nanocatalyst was determined using various techniques such as SEM, TEM, FT-IR, EDS, ICP-OES, VSM, N_2_ adsorption–desorption isotherms, XRD, and TGA. In both of the mentioned coupling paths, the yields of the products were very favorable and ranged from 90 to 98%. Also, they had significant features compared to previous reports, such as very short reaction time (5–15 and 7–20 min respectively in the Suzuki and Stille reactions), easy work-up, broad substrate scope, ease of separation of the catalyst using a magnet, suitable reproducibility of the catalyst in 6 runs, heterogeneous nature of the catalyst and not washing it during consecutive runs with confirmation of hot filtration and ICP-OES methods.

## Introduction

Creating reusable and environmentally friendly catalytic systems has received a lot of interest in recent years^1–3^. In this context, there is a great deal of interest in a range of chemical reactions related to the immobilization of catalysts on solid materials^4–6^. Nanoparticles, among other solid materials, are suitable supports and play essential roles in the creation of catalysts in terms of recyclability and selectivity^7,8^. Because of their high surface-to-volume ratio, outstanding efficiency, stability, and recoverability, nanomaterials can be used as a bridge between homogeneous and heterogeneous catalysis^9–11^. Iron oxide magnetic nanoparticles, in particular Fe_3_O_4_ nanoparticles, have piqued the interest of researchers due to their distinctive features and application possibilities in a variety of areas and sciences^12,13^. The most notable advantage of magnetic nanoparticles is their ease of separation from reaction mixtures using an external magnet, which increases adaptability in work-up operations^14,15^.

Mesoporous silica materials, such as SiO_2_, on the other hand, have drawn a lot of interest in the domains of adsorption^16^, drug delivery systems^17^, extraction^18^, and particularly catalysis^19^. Their unique qualities, such as their substantial surface area, result in significant loading capacities for catalyst immobilization. Additionally, their large and uniform pore size is appropriate for immobilizing organic ligands and transition metals, and their excellent thermal stability (more than 800 °C) makes them usable in difficult reaction circumstances^20,21^. These characteristics urge scientists to utilize these substances as supports for various catalyst types. For practical applications, the coating of Fe_3_O_4_ particles with SiO_2_ layers to create porous magnetic nanocomposite is of tremendous interest. Because mesoporous magnetic nanocomposites offer the benefits of mesoporous materials (such as large surface area and large pore size) and magnetic nanoparticles (such as easy separation using an external magnet). To achieve excellent selectivity and activity, as well as to facilitate simple catalyst separation and reusability, homogeneous catalysts are immobilized into mesoporous magnetic nanocomposite^20–24^. Solid-state synthesis processes allow for precise control over the size, shape, and morphology of the nanoparticles. This control is crucial in catalysis as the activity and selectivity of a catalyst are often size-dependent^25–27^. Nanocatalysts synthesized using solid-state methods often exhibit enhanced catalytic activity compared to their bulk counterparts. The high surface-to-volume ratio of nanoparticles allows for more active sites, leading to increased catalytic efficiency^25–29^. Supported catalysts on solid substrates are often easier to handle and recover from reaction mixtures. The solid substrate provides mechanical support and facilitates catalyst separation and recycling, making the catalytic process more efficient and cost-effective^25–29^.

One of the most significant processes in the synthesis of organic compounds is carbon–carbon cross-coupling reactions^30^. Palladium has been the most widely used transition metal catalyst for various coupling reactions during the last few decades. Although homogeneous palladium catalysts like Pd(OAc)_2_ and PdCl_2_ have generally high activity, their use has been constrained by their inability to be easily separated from the reaction mixture and recovered, as well as by the considerable environmental damage they cause^31–35^. The creation of heterogeneous palladium catalysts to overcome the aforesaid limitations has important theoretical and practical significance and also is one of the key aims of green chemistry^36–38^. Consequently, to immobilize the palladium catalysts, solid materials such as polymer materials, carbon, zeolite, and various forms of silica (amorphous silica, mesoporous molecular sieves, solids produced by co-condensation of silicate precursors, and many more) have been utilized^39–43^.

Carbon–carbon couples have wide-ranging applications across industries such as pharmaceuticals, materials science, and chemical manufacturing. They are employed in drug development to design and synthesize medicinal compounds with specific biological activities^44^. In polymer synthesis, carbon–carbon bonds are vital for determining the strength, flexibility, and other properties of polymers^45,46^. Organic synthesis extensively utilizes carbon–carbon coupling reactions to construct complex organic molecules using cross-coupling reactions. Several carbon–carbon coupling reactions, such as the Suzuki–Miyaura, Stille, Heck, and Negishi reactions, are commonly utilized in chemical manufacturing. These reactions enable the formation of carbon–carbon bonds between different organic fragments, facilitating the assembly of complex molecules required for specific applications^47,48^. Additionally, carbon–carbon composites, comprising carbon fibers within a carbon matrix, are highly robust and lightweight materials used in aerospace and automotive industries for manufacturing aircraft parts and sports car bodies^49,50^.

In this scientific article, considering the importance of carbon–carbon couples in different industries, we present the synthesis and characterization of a novel anchored palladium complex on the BisPyP@Bilayer-SiO_2_@NMP organic–inorganic hybrid (Pd-BisPyP@bilayer-SiO_2_@NMP) as an effectual mesoporous magnetic catalyst for the cross-coupling reactions in the presence of phenylboronic acid and triphenyltin chloride in very short periods under relatively mild conditions.

## Experimental

### Chemicals and devices

All specifications of raw materials and devices are given in the related files.

### Preparation of the catalyst

**Scheme 1:**
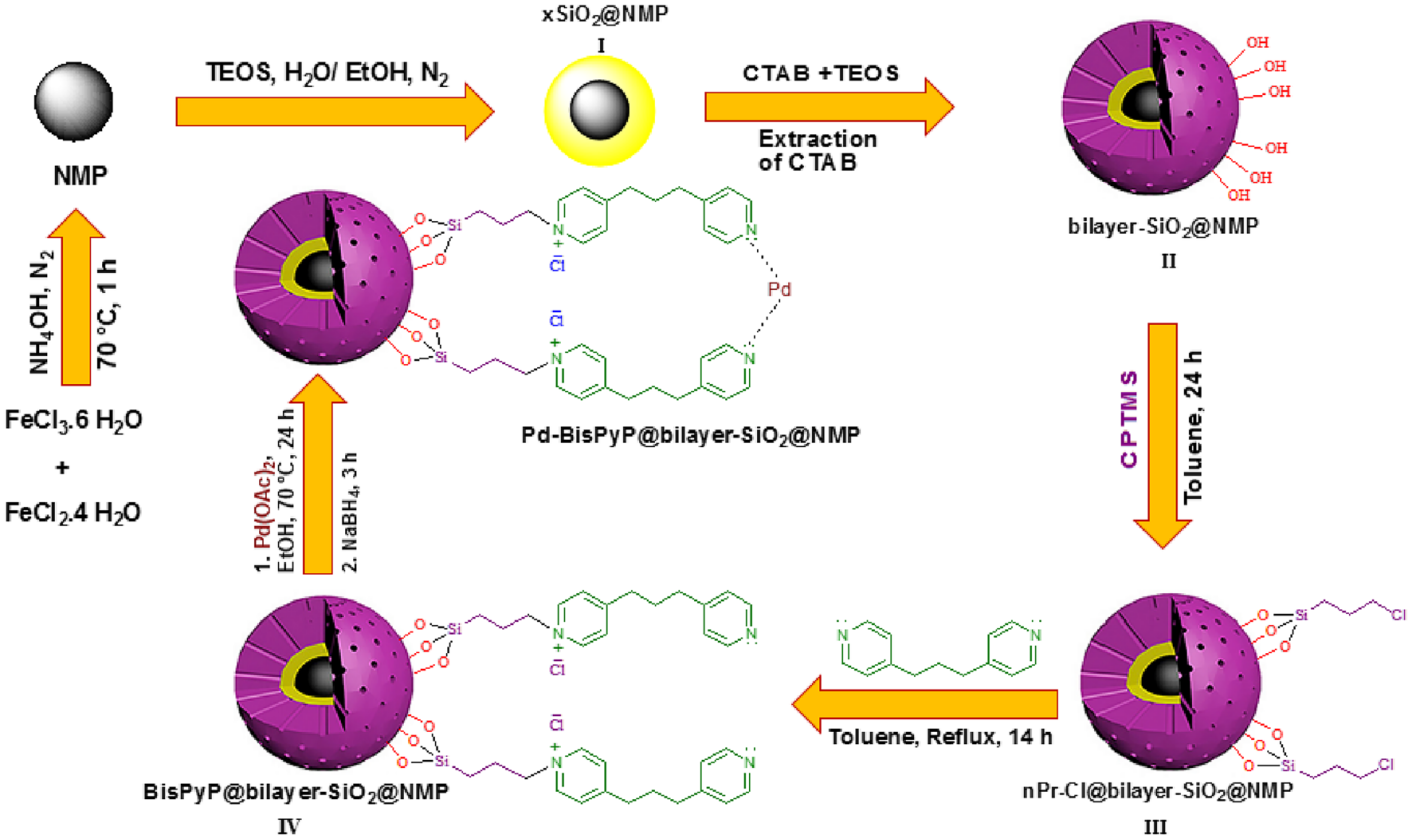
The synthesis steps Pd-BisPyP@bilayer-SiO_2_@NMP.

In order to produce the Pd-BisPyP@bilayer-SiO_2_@NMP catalyst, we first created Fe_3_O_4_ magnetic nanoparticles using the co-precipitation approach and intermediate **I** (xSiO_2_@Fe_3_O_4_) according to prior research^51^. In continuation, in order to synthesize Fe_3_O_4_ coated with two layers of silica (bilayer-SiO_2_@Fe_3_O_4_), intermediate **I** (0.1 g) was dispersed in a mixed solution of deionized water (80 mL), cetyltrimethylammonium bromide (CTAB, 0.3 g), aqueous ammonia (25 wt%, 1 mL), and ethanol (60 mL) via ultrasonication (25 min). The next step was to mix a suspension containing 0.2 g of intermediate **II**, 1.66 mM of 3-chloropropyltriethoxysilane, and 25 mL of dry toluene solvent under reflux for 24 h. After the end of the reflux time to produce intermediate **III**, the resulting precipitate was filtered and washed with ethanol, toluene and deionized water. The 1,3-bis(4-pyridyl)propane was then immobilized on its surface by stirring a combination of 1,3-bis(4-pyridyl)propane (0.1 g), intermediate **III** (0.3 g), and toluene (25 mL) for 14 h while it was refluxed in an N_2_ gas atmosphere (Intermediate **IV**). The final stage involved dispersing Intermediate **IV** (0.5 g) in ethanol and combining it with 0.25 g of palladium acetate that refluxed for 24 h. After that, the NaBH_4_ (0.4 mmol) was added to the reaction mixture and stirred for 3 h. The black solid product Pd-BisPyP@bilayer-SiO_2_@NMP was obtained by filtration, washed with ethanol and dried in oven at 70 °C (Scheme 1).

### General method for carbon–carbon coupling through Suzuki or Stille reactions

1 mmol aryl halide, 1 mmol phenylboronicacid (or 0.5 mmol triphenyltin chloride), 3 mmol potassium hydroxide and 5 mg Pd-BisPyP@bilayer-SiO_2_@NMP (1.06 mol%) were stirred in a flask at a temperature of 80 °C in PEG-based solvent (3 mL) until the reaction was completed by TLC monitoring. After the time required to complete the reaction, the catalyst was separated by simple filtration in the vicinity of an external magnetic field. It was cleaned with EtOAc and dried in oven for another run. Next, the organic layer was extracted with diethyl ether (5 mL, four times) from PEG in a separating funnel by help of adding water. Finally, the solvent was evaporated and pure biphenyl derivatives were obtained in excellent yields.

### Ethics approval and consent to participate

The author's declare that the paper is not be submitted simultaneously to another journal. The submitted work is original and has not been published elsewhere in any form or language, and the authors have no conflict of interest regarding this manuscript. The authors agree to participate in submitting our manuscript to this journal, and agree to the publication of our research data in this journal.

## Results and discussion

**Figure 1:**
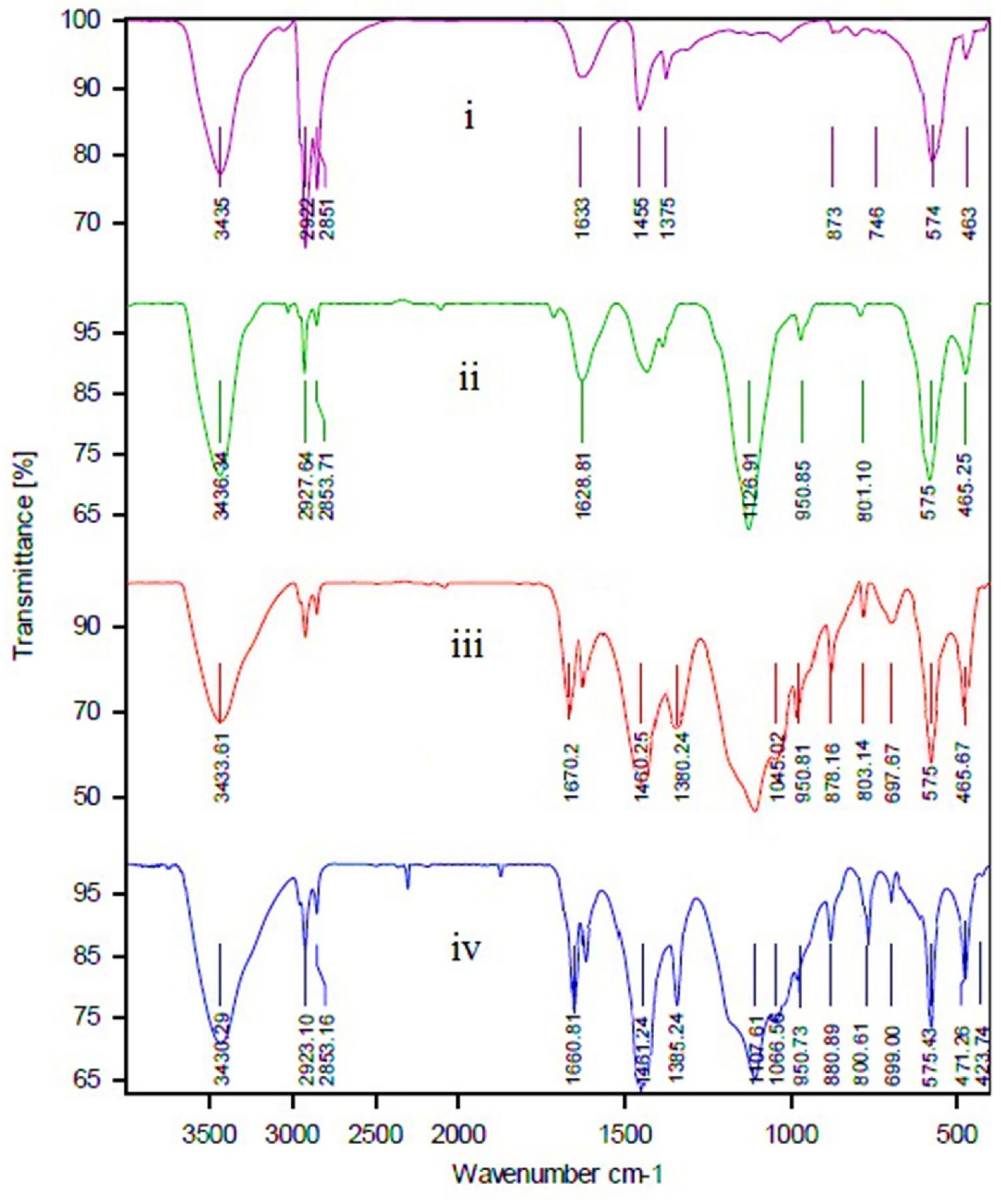
FT-IR spectra of NMP (Fe_3_O_4_) (i), bilayer-SiO_2_@NMP (ii), BisPyP@bilayer-SiO_2_@NMP (iii), and Pd-BisPyP@bilayer-SiO_2_@NMP (iv).

The successful functionalization of the bare Fe_3_O_4_ nanoparticles can be deduced from the FT-IR technique. The FT-IR spectra for NMP (Fe_3_O_4_) (i), bilayer-SiO_2_@NMP (ii), BisPyP@bilayer-SiO_2_@NMP (iii), and Pd-BisPyP@bilayer-SiO_2_@NMP (4), are demonstrated in Fig. 1. In Fig. 1 i, the two peaks at 463 and 574 cm^−1^ are attributed to the vibrations of Fe–O bond^52^. In the FT-IR pattern of bilayer-SiO_2_@NMP, the coating of bare Fe_3_O_4_ nanoparticles with silica layers was confirmed by observing the absorption band related to Si–O–Fe vibration at 950 cm^−1^^52^. Also, the 803 and 1026 cm^−1^ bands are corresponded to the symmetric and asymmetric vibrations of Si–O–Si in SiO_2_ (Fig. 1ii)^52^. In Fig. 1iii, the presence of immobilized 3-chloropropyltriethoxysilane (nPr-Cl@bilayer-SiO_2_@NMP), and the grafting 1,3-bis(4-pyridyl)propane to intermediate **III** are confirmed via Si–C stretching vibration at 1045 cm^−1^^53^, and C–N stretching vibration at 1380 cm^−1^, respectively^53^. Additionally, the anchoring of palladium to BisPyP@bilayer-SiO_2_@NMP was confirmed with a shift of C=N vibration stretching in Pd-BisPyP@bilayer-SiO_2_@NMP (1660 cm^−1^ in Fig. 1iv), compared to BisPyP@bilayer-SiO_2_@NMP (1670 cm^−1^ in Fig. 1iii), to a lower frequency which is the reason for this shift is the coordination of Pd to supported BisPyP onto functionalized bilayer-SiO_2_@NMP^21^. Other adsorptions in the structure of the catalyst are mentioned in Table 1.

**Table 1:** FT-IR data of NMP (Fe_3_O_4_) (i), bilayer-SiO_2_@NMP (ii), BisPyP@bilayer-SiO_2_@NMP (iii), and Pd-BisPyP@bilayer-SiO_2_@NMP (iv).

Absorption (cm^−1^)	Related bond
574 (i), 575 (ii), 575 (iii), 576 (iv)	Fe–O stretching^52^
803 (ii), 801 (iii), 800 (iv)	Si–O–Si symmetric stretching^52^
950 (ii), 950 (iii), 950 (iv)	Si–O–Fe stretching^52^
1045 (iii), 1066 (iv)	Si–C Stretching^53^
1126 (ii), 1100 (iii), 1107 (iv)	Si–O–Si asymmetric stretching^52^
1380 (iii), 1385(iv)	C–N stretching^53^
1670 (iii), 1660 (iv)	C=N stretching^21^
1460 (iii), 1461 (iv)	C=C stretching^21^
1633 (i), 1628 (ii), ~ 1632 (iii), ~ 1630 (iv)	OH bending on the surface of the SiO_2_ and Fe_3_O_4_^52^
2923 (iii), 2923 (iv)	C–H symmetric stretching^53^
3435 (i), 3436 (ii), 3433 (iii), 3430 (iv)	OH stretching on the surface of the SiO_2_ and Fe_3_O_4_^52,53^

**Figure 2:**
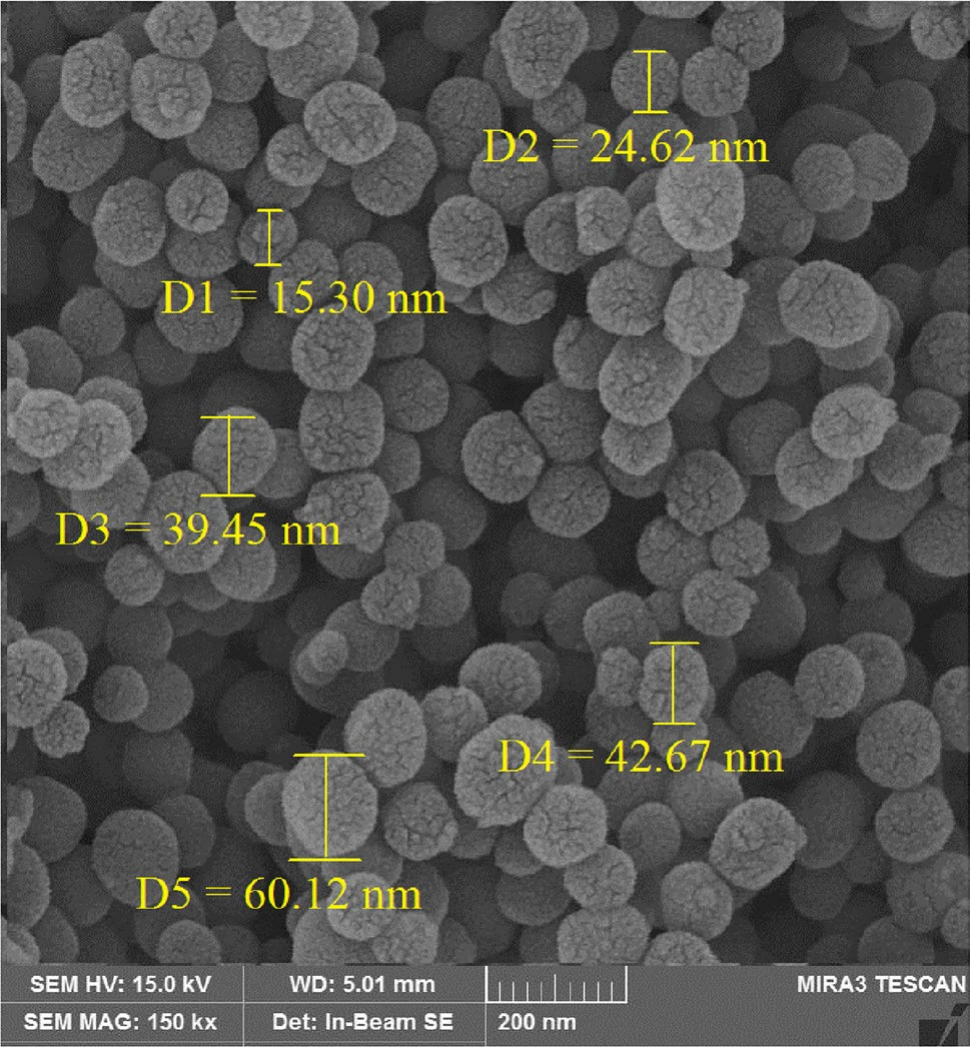
The SEM image of Pd-BisPyP@bilayer-SiO_2_@NMP.

SEM was used to analyze the surface morphology of the Pd-BisPyP@bilayer-SiO_2_@NMP nanocatalyst. Quasi-spherical particles with an average size of 15 to 60 nm can be seen in the SEM image of Pd-BisPyP@bilayer-SiO_2_@NMP (Fig. 2).

**Figure 3:**
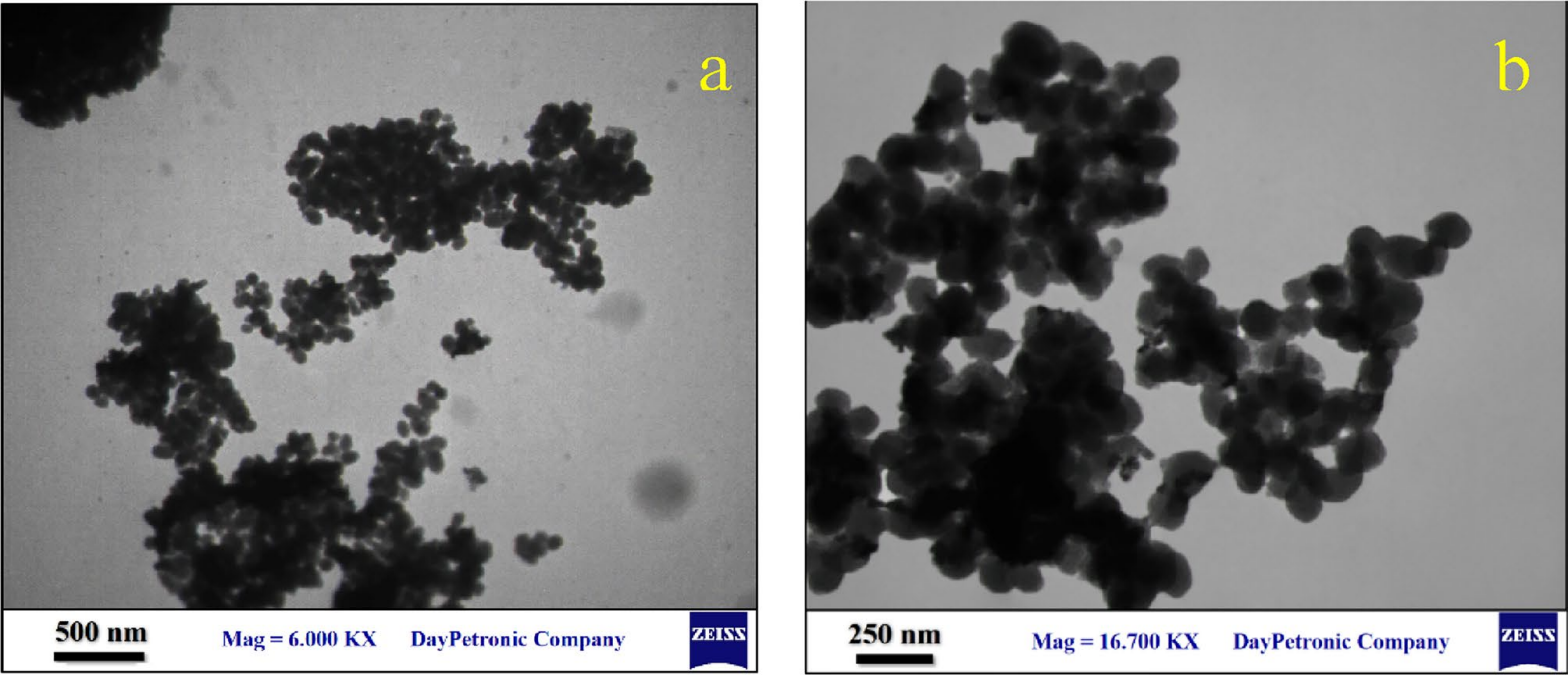
TEM images of BisPyP@bilayer-SiO_2_@NMP (**a**), and Pd-BisPyP@bilayer-SiO_2_@NMP (**b**).

The study of the TEM images of BisPyP@bilayer-SiO_2_@NMP (a), and Pd-BisPyP@bilayer-SiO_2_@NMP (b) depicted in Fig. 3 amply supports the conclusions drawn from the SEM image, because the quasi-spherical shape of the nanoparticles can be clearly seen in these images. In addition, the comparison of these two images shows that the quasi-spherical structure of the substrate nanoparticles has not undergone much change after anchoring palladium on it.

**Figure 4:**
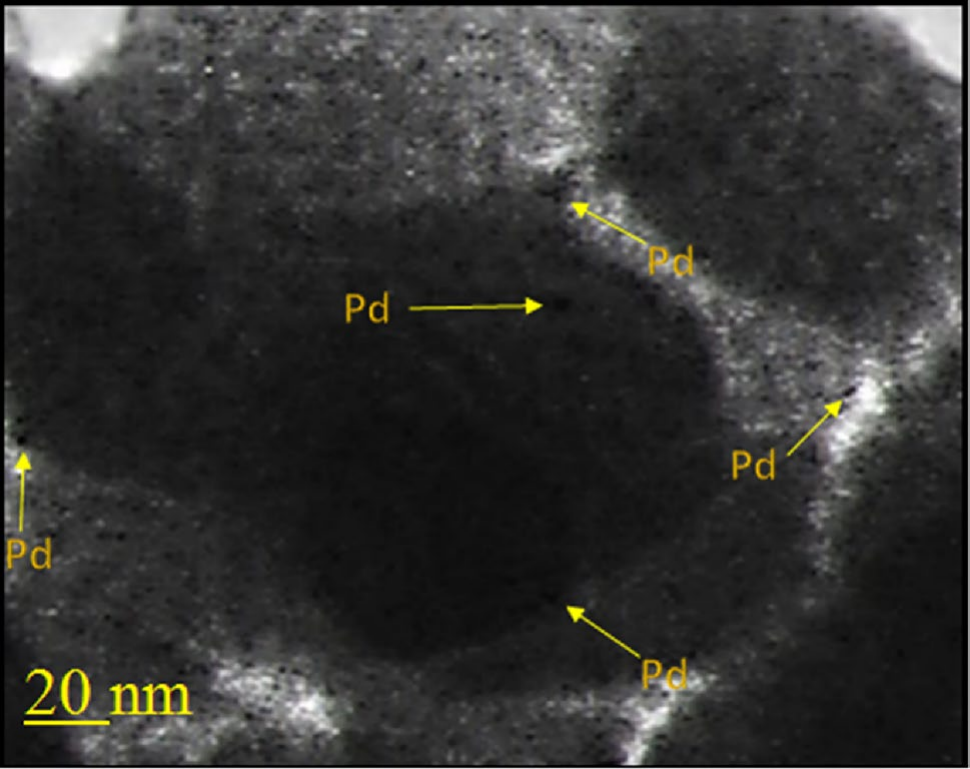
HRTEM image of Pd-BisPyP@bilayer-SiO_2_@NMP.

In another study, the structure of the catalyst was reviewed with a high-resolution TEM image (Fig. 4). This image supports the distribution of Pd particles throughout the support, which can be seen as black dots.

**Figure 5:**
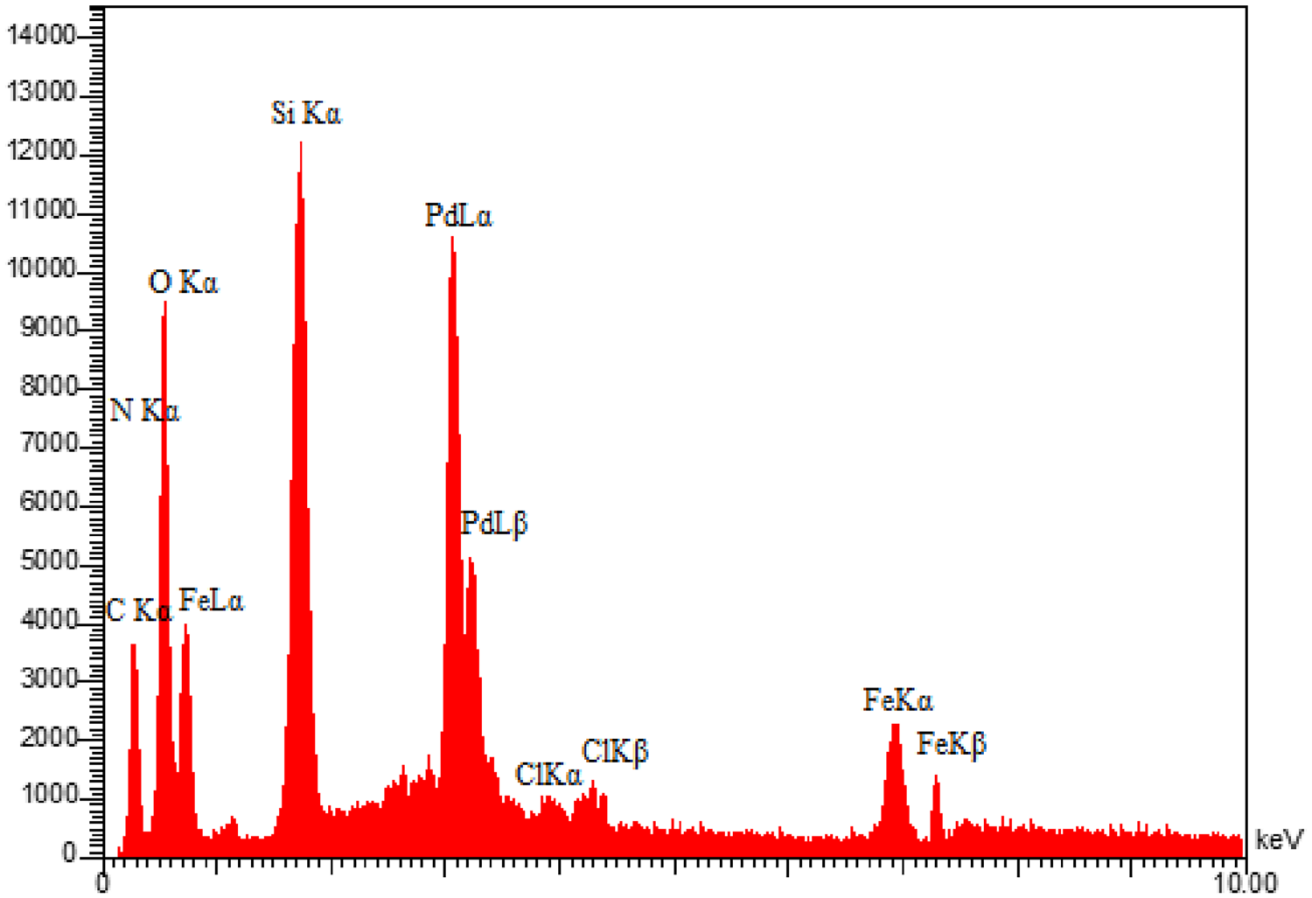
The EDS spectrum of Pd-BisPyP@bilayer-SiO_2_@NMP.

**Figure 6:**
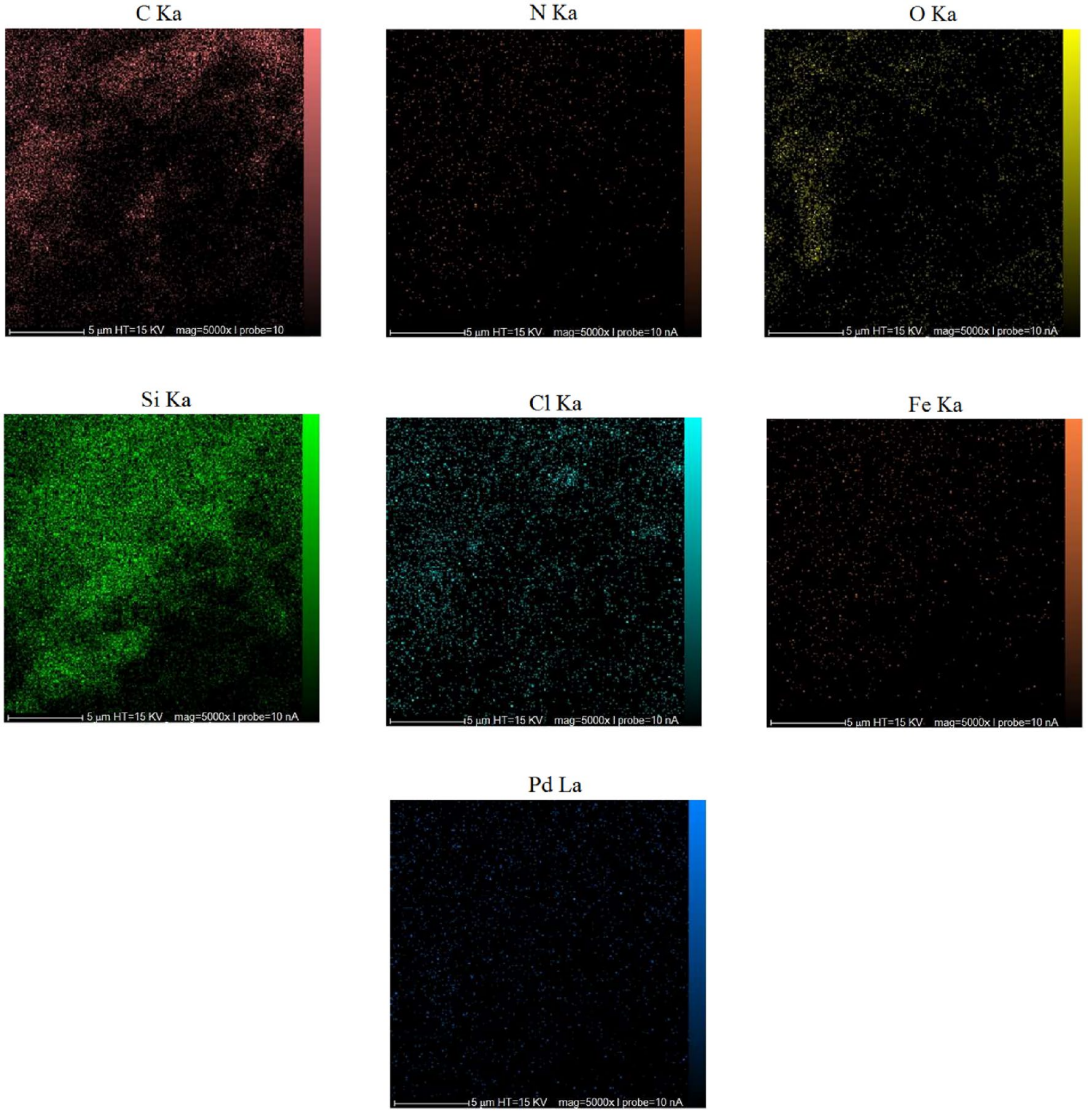
The elemental mapping images of Pd-BisPyP@bilayer-SiO_2_@NMP.

Energy dispersive X-ray spectroscopy analysis, often called EDS, is a practical technique for revealing information on different catalyst elements. Figure 5 confirms the successful fabrication of nanoparticles by showing the presence of C, O, N, Fe, Si, Cl and Pd species in the Pd-BisPyP@bilayer-SiO_2_@NMP nanocatalyst structure. Meanwhile, the elemental mapping technique images support the presence of all the mentioned constituent elements in the mesoporous skeleton (Fig. 6). It is worth noting, using inductively coupled plasma atomic emission spectroscopy, the precise quantity of palladium in Pd-BisPyP@bilayer-SiO_2_@NMP was determined to be 2.123 × 10^–3^ mol g^−1^.

**Figure 7:**
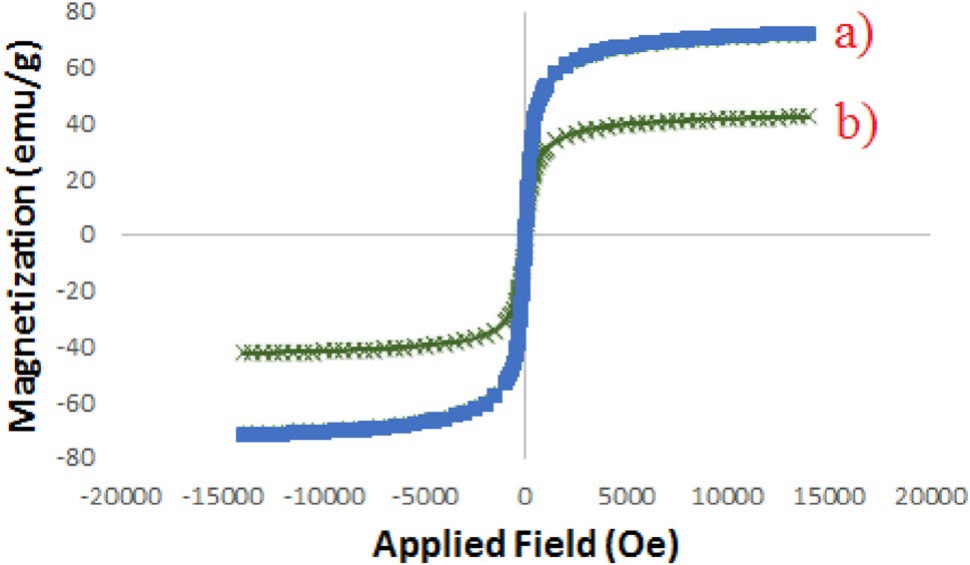
Magnetization curves for Fe_3_O_4_ (**a**), and Pd-BisPyP@bilayer-SiO_2_@NMP (**b**).

Magnetic measurement for bare Fe_3_O_4_ (a) and Pd-BisPyP@bilayer-SiO_2_@NMP (b) were carried out, and results are demonstrated in Fig. 7. According to this figure, the saturation magnetic value of catalyst (44.61 emu g^−1^) is lower than bare Fe_3_O_4_ (73.84 emu g^−1^). This difference could be attributed to the presence of silica layers and the Pd-BisPyP complex attached to the nanoparticle surface. However, by applying an external magnetic field, the catalyst can be rapidly separated from the reaction mixture.

**Figure 8:**
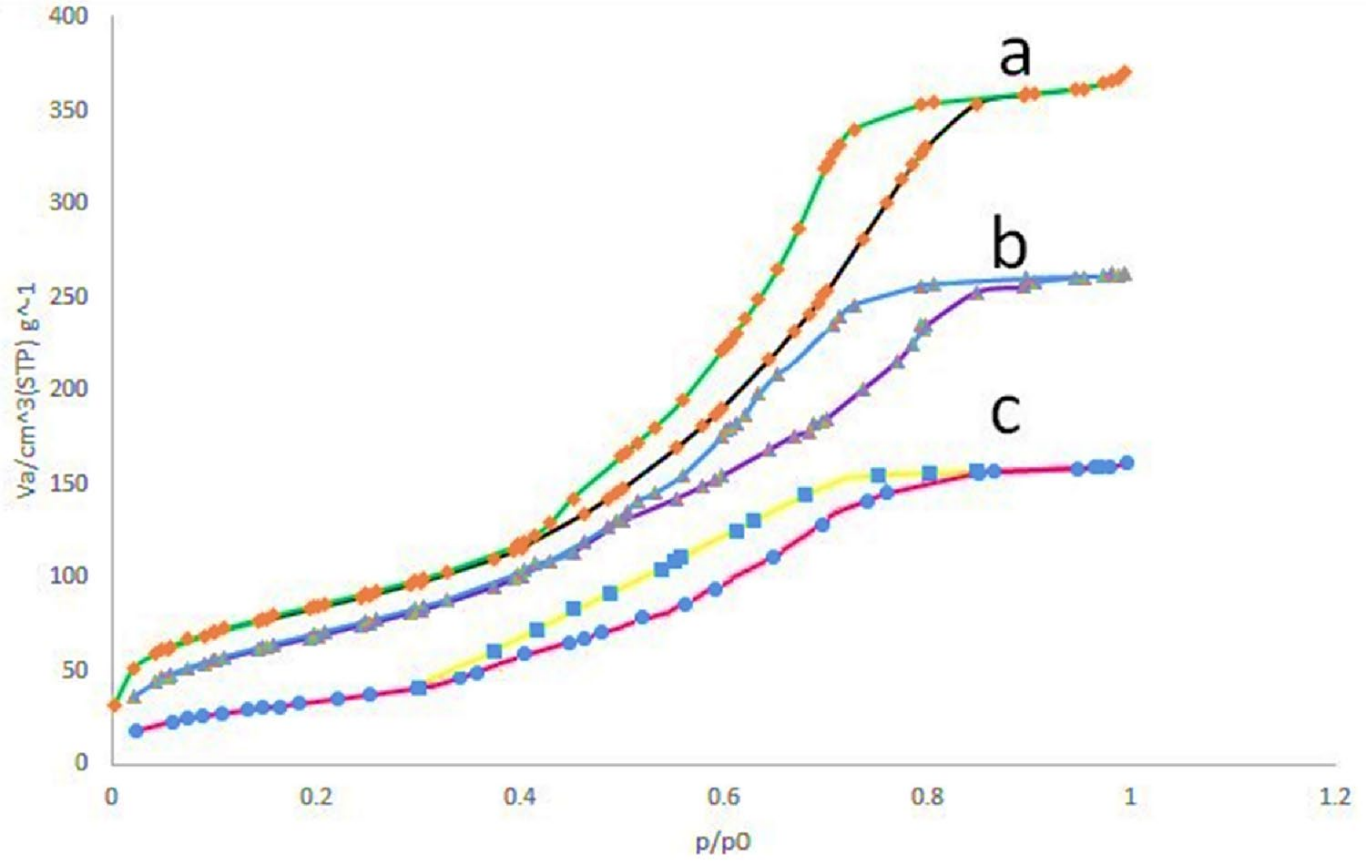
Nitrogen adsorption–desorption isotherms of xSiO_2_@Fe_3_O_4_ (**a**), BisPyP@bilayer-SiO_2_@NMP (**b**), and Pd-BisPyP@bilayer-SiO_2_@NMP (**c**).

Investigating the porosity of the Pd-BisPyP@bilayer-SiO_2_@NMP nanocatalyst and its generating components was done using the nitrogen adsorption–desorption technique (Fig. 8). In Table 2, the findings of this investigation are enumerated. According to the information in this table, Pd-BisPyP@bilayer-SiO_2_@NMP has a lower specific surface area than BisPyP@bilayer-SiO_2_@NMP, xSiO_2_@Fe_3_O_4_, and Fe_3_O_4_; this is because organic groups and Pd complexes have been anchored on the mesoporous channels of magnetic silica nanoparticles. In addition, the pore size distribution (BJH model) and BET curves of the catalyst are provided in the supplementary material (Figs. S7 and S8, respectively).

**Table 2:** Texture properties of Pd-BisPyP@bilayer-SiO_2_@NMP.

Sample	SBET (m^2^/g)	r_p,peak_ by BJH method (nm)^a^	Pore vol (cm^3^/g)	Ref
Fe_3_O_4_	480.0	1.254	0.803	^51^
xSiO_2_@Fe_3_O_4_	463.3	1.551	0.779	–
BisPyP@bilayer-SiO_2_@NMP	410.9	1.883	0.722	–
Pd-BisPyP@bilayer-SiO_2_@NMP	327.7	2.088	0.645	–

^a^r_p,peak_: Mean pore radius.

**Figure 9:**
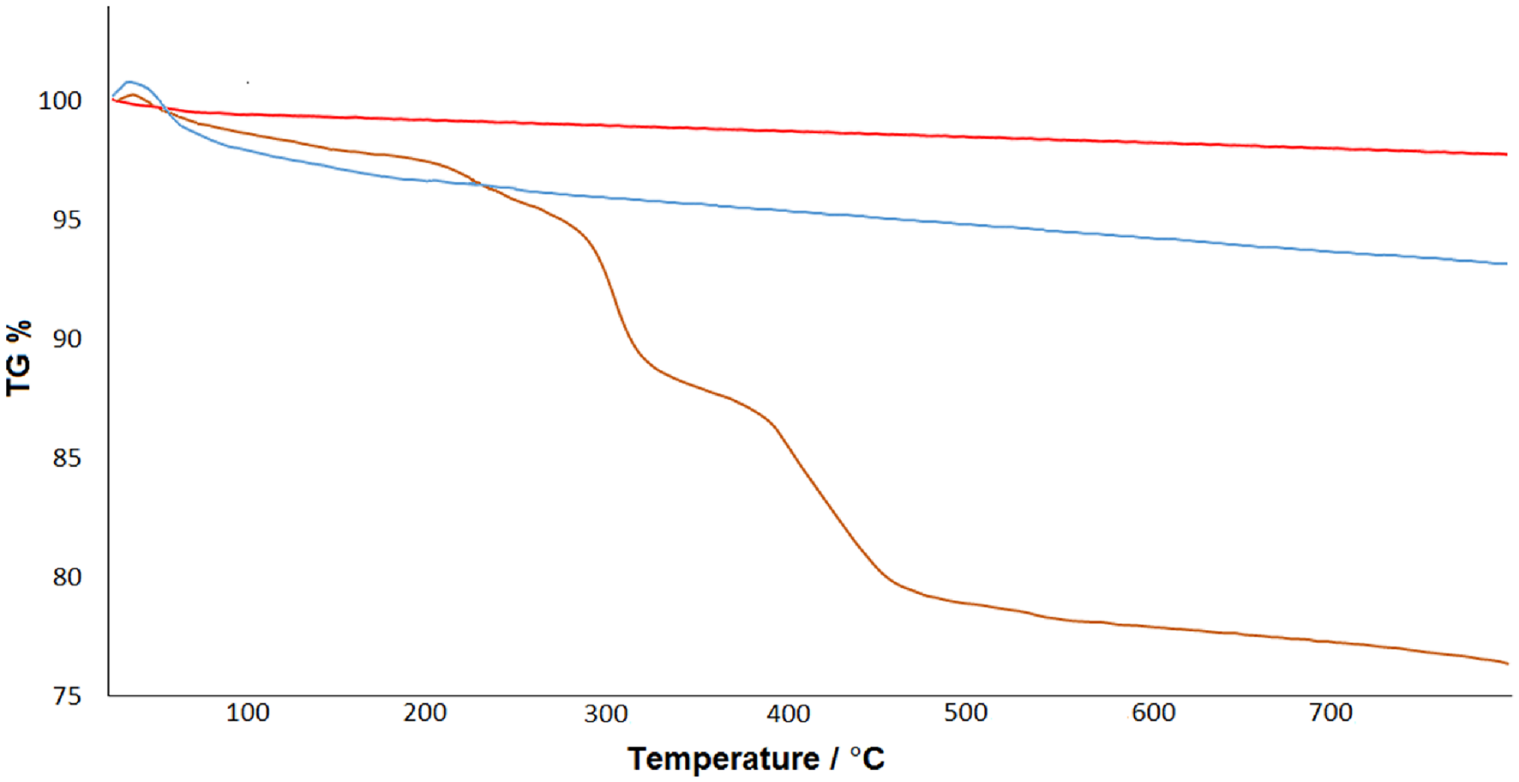
TGA curves of bare NMP (red curve), bilayer-SiO_2_@NMP (blue curve), and Pd-BisPyP@bilayer-SiO_2_@NMP (green curve).

Thermal gravimetric analysis (TGA) was used to infer the thermal stability of the produced nanocatalyst and the formation of bonds between the complex and the nanoparticles. Figure 9 displays the TGA curves for bare NMP (green curve), bilayer-SiO_2_@NMP (blue curve), and the Pd-BisPyP@bilayer-SiO_2_@NMP (red curve). The TGA diagram of all three samples shows a slight weight loss below 200 °C, which is caused by the removal of surface hydroxyl groups and physically absorbed solvents. The TGA curve of Pd-BisPyP@bilayer-SiO_2_@NMP showed two other stages of weight loss in addition to the preliminary weight loss (3.09% weight loss at temperature below 200 °C): (i) 10.72% weight loss in the temperature range of 200–380 °C (arising from decomposition of organic moieties of the complex), and (ii) 9.83% decrease weight between 380 and 700 °C (due to the decomposition of silanol groups)^54^. The above observations confirmed the thermal stability of the catalyst and the immobilization of the palladium complex on the bilayer-SiO_2_@NMP.

**Figure 10:**
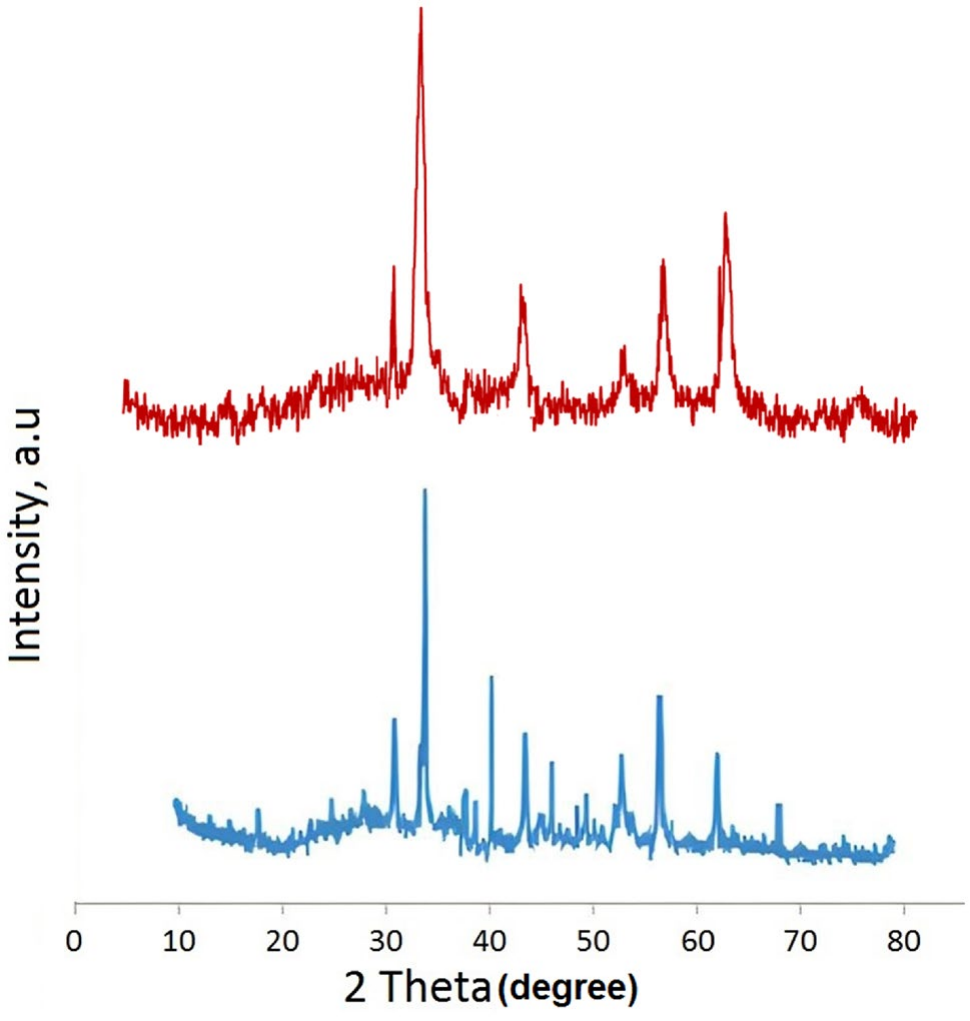
XRD patterns of bare NMP (crimson pattern), and Pd-BisPyP@bilayer-SiO_2_@NMP (blue pattern).

The structural characteristics of bare NMP (crimson pattern), and Pd-BisPyP@bilayer-SiO_2_@NMP (blue pattern) were investigated using the XRD method in Fig. 10. The XRD pattern of these two samples, which consists of many peaks at 2θ = 30.66° (220), 34.50° (311), 43.33° (400), 52.66° (422), 56.11° (511) and 62.02° (440), which are related to the crystallographic faces of Fe_3_O_4_ particles^55^. Furthermore, the existence of Pd in the catalyst structure was confirmed by the Bragg angles at = 40.01° (111), 46.29° (200), and 68.01° (220) (blue pattern)^56^. It is worth noting that the broad peak at 2θ = 20–30°, and also the peaks observed at 2θ = 18.01°, 48.65°, 49.33°, 37.33° and 38.41° indicate the presence of amorphous silica around the Fe_3_O_4_ particles and the crystalline nature of the catalyst, respectively^54^.

### Examine of the catalytic property

**Figure 11:**
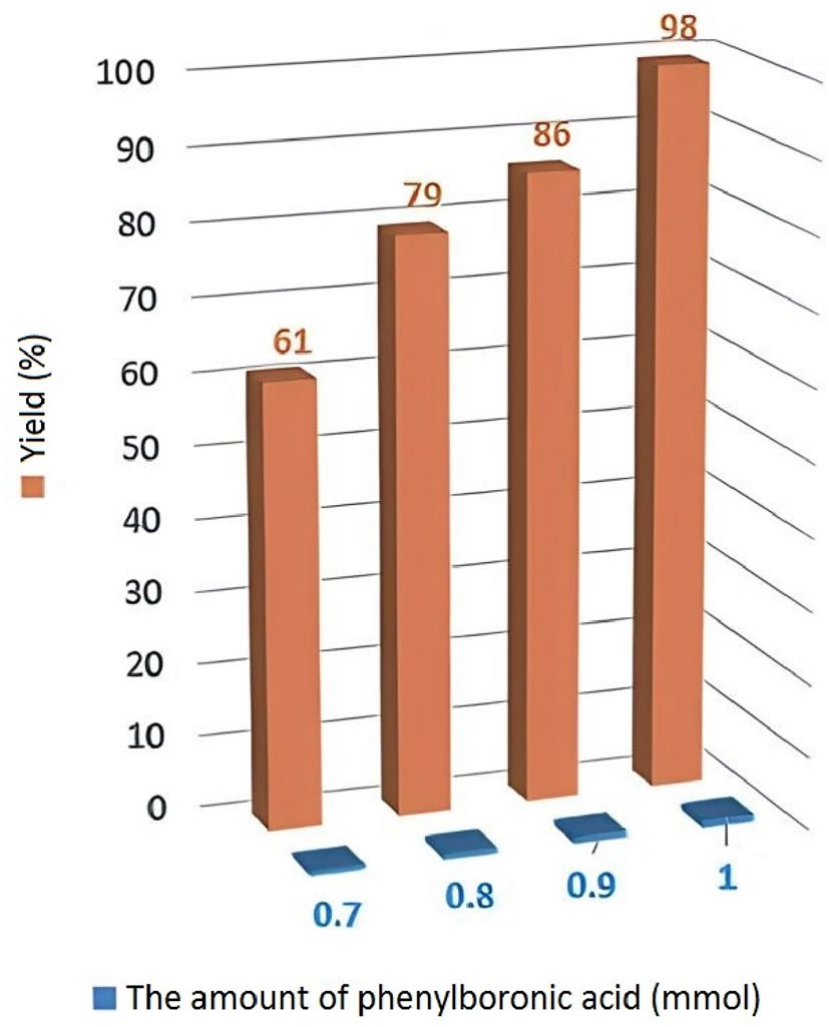
Effect of amount of phenylboronic acid on the efficiency of the model reaction.

The catalytic performance of Pd-BisPyP@bilayer-SiO_2_@NMP in the C–C cross-coupling reaction of aryl halides with phenylboronic acid and triphenyltin chloride was carefully studied. In this regard, to determine the optimize conditions, the reaction between iodobenzene and phenylboronic acid was chosen as a model, and the effect of various factors, including temperature, amount of catalyst, solvent, and base on it was monitored. The results are shown in Table 3. In the first step, the effect of the nature of the solvent on the reaction of the model was studied. The results showed that the type of solvent used has a significant impact on the progress of the reaction, so that no product was observed in 1,4-dioxane (Table 3, entry 1), and small amounts of the product were obtained in H_2_O and EtOH in the presence of 5 mg of catalyst (Table 3, entries 2 and 3). However, in solvents such as DMF (dimethylformamide) and DMSO (dimethyl sulfoxide), the corresponding biphenyl product was isolated with 69 and 72 yields, respectively (Table 3, entries 4 and 5), and the highest yield was observed in PEG-400 solvent (Table 3, entry 6). Therefore, the PEG-400 solvent was chosen as the most suitable solvent for the mentioned reaction. In the second step, we studied the effect of different bases on the model's reaction. These bases included organic bases (DMAP, Et_3_N), monobasic (KOH, NaOH), dibasic (Na_2_CO_3_), and NaHCO_3_ (Table 3, entries 6–11). The results showed that Na_2_CO_3_ can be a highly effective base for the Suzuki cross-coupling reaction (Table 3, entry 6). Also, KOH and NaOH, despite being stronger bases than Na_2_CO_3_ based on their known K_b_ values, ultimately did not lead to a significant yield of the product, because according to the studies of Knecht and his colleagues^57^, for monobasic systems (KOH, NaOH), the amount of free and active hydroxide compared to Na_2_CO_3_ and NaHCO_3_ is more, and this causes their strong combination with palladium species and decrease in biphenyl efficiency for cross-coupling reaction. Also, the reason for the ineffectiveness of organic bases such as Et_3_N and DMAP is their weak play power. The result of this study, as well as the failure to perform the reaction in the absence of base (Table 3, entry 12), proved that an average basic capacity is necessary to carry out the mentioned reaction using Pd-BisPyP@bilayer-SiO_2_@NMP. In the third step, our studies were focused on the effect of temperature on the progress of the reaction (Table 3, entries 6 and 13–15). Examining the result of the model reaction in the temperature range of 25 to 90 °C reflected the fact that the reaction is sensitive to temperature, so that a higher temperature can shorten the reaction time and cause a better progress of the reaction. Further investigations showed that the best temperature for the reaction progress is 70 °C (Table 3, entry 6). In the fourth step, the impact of the amount of phenylboronic acid on the performance was also studied (Fig. 11). The reactions performed in the presence of 0.7, 0.8, 0.9, and 1.0 mmol of phenylboronic acid provide 61, 79, 86, and 98 isolated yields, respectively. It has been determined that 1 mmol of phenylboronic acid with 1 mmol of iodobenzene are the optimal amounts to complete the reaction. In the last step, the model reaction was checked with quantities of 4, 5 and 6 mg of Pd-BisPyP@bilayer-SiO_2_@NMP because the coupling reaction does not occur under catalyst-free conditions (Table 3, entry 18). It was found that 5 mg of BisPyP@bilayer-SiO_2_@NMP is the optimal amount (Table 3, entry 6), while reducing the amount of catalyst to 4 mg in 60 min resulted in lower efficiency (Table 3, entry 16), increasing the amount of its catalyst to 6 mg did not show superiority over the optimal state (Table 3, entry 17).

**Table 3:** C–C coupling of iodobenzene with phenylboronic acid in the presence of Pd-BisPyP@bilayer-SiO_2_@NMP under various conditions^a^.


Entry	Solvent	Base (mmol)	Temperature (°C)	Catalyst (mg)	Time (min)	Yield (%)^b^
1	1,4-Dioxane	Na_2_CO_3_	70	5	5	–
2	EtOH	Na_2_CO_3_	Reflux	5	5	26
3	H2O	Na_2_CO_3_	70	5	5	22
4	DMSO	Na_2_CO_3_	70	5	5	69
5	DMF	Na_2_CO_3_	70	5	5	72
6	PEG	Na_2_CO_3_	70	5	5	98
7	PEG	Et_3_N	70	5	5	24
8	PEG	DMAP	70	5	5	27
9	PEG	KOH	70	5	5	64
10	PEG	NaOH	70	5	5	47
11	PEG	NaHCO_3_	70	5	5	85
12	PEG	–	70	5	240	—
13	PEG	Na_2_CO_3_	25	5	180	—
14	PEG	Na_2_CO_3_	50	5	60	57
15	PEG	Na_2_CO_3_	90	5	5	98
16	PEG	Na_2_CO_3_	70	4	60	79
17	PEG	Na_2_CO_3_	70	6	5	98
18	PEG	Na_2_CO_3_	70	–	1440	–

^a^Reaction conditions: iodobenzene (1 mmol), phenylboronic acid (1 mmol), base (3 mmol), solvent (2 mL).

^b^Isolated yield.

After determining the best conditions for the coupling of iodobenzene with phenylboronic acid, in another study this reaction was carried out in the presence of the catalyst parents, i.e. NMP (Fe_3_O_4_) (Table 4, entry 1), bilayer-SiO_2_@NMP (Table 4, entry 2), BisPyP@bilayer-SiO_2_@NMP (Table 4, entry 3), and also Pd-BisPyP@xSiO_2_@NMP (The only difference of this catalyst compared to Pd-BisPyP@bilayer-SiO_2_@NMP is the use of a silica layer (not two layers) around Fe_3_O_4_) (Table 4, entry 4). Subsequently, the amount of palladium in the Pd-BisPyP@bilayer-SiO_2_@NMP and Pd-BisPyP@xSiO_2_@NMP measured via the ICP technique, which was equal to 2.123 × 10^−3^ and 1.112 × 10^−3^ mol g^−1^, respectively. Experimental evidence from the ICP technique and Table 4 demonstrated that trick of utilizing two layers of SiO_2_ on NMP, and also anchoring palladium on the BisPyP@bilayer-SiO_2_@NMP was effective for the better progress of the reaction, because the reaction failed in the presence of the catalyst's parents (Table 4, entries 1–3), and only when they connect to each other and form the catalyst, a strong synergistic effect of the catalyst is observed. Immobilization of two layers of SiO_2_ on NMP leads to the bonding of organic groups to the surface of the substrate, and this causes more palladium to enter the mesoporous channels, and complete the reaction with less amount of catalyst. Besides, the use of double-layer silica prevents the aggregation of magnetic nanoparticles.

**Table 4:** Investigating the influence of NMP, bilayer-SiO_2_@NMP, BisPyP@bilayer-SiO_2_@NMP, and Pd-BisPyP@xSiO_2_@NMP in the coupling of iodobenzene with phenylboronic acid as a model compound under optimized conditions.

Entry	Catalyst	Yield (%)^a^
1	NMP (Fe_3_O_4_)	–^b^
2	bilayer-SiO_2_@NMP	–^b^
3	BisPyP@bilayer-SiO_2_@NMP	–^b^
4	Pd-BisPyP@xSiO_2_@NMP	73

^a^Isolated yield under optimized conditions.

^b^No reaction.

To find out the scope of the catalytic system under examination, cross-coupling of phenylboronic acid with diverse aryl halides was tested. The results showed that all reactions proceed at high speed to obtain the corresponding biphenyl derivative with excellent efficiency (Table 5). The electron-donating or electron-withdrawing properties of the substituents do not seem to have a significant effect on the product yield. Also, Aryl bromides and aryl iodides provide better results than aryl chlorides.

**Table 5:** Preparation of biphenyl derivative from Suzuki reaction using Pd-BisPyP@bilayer-SiO_2_@NMP^a^.


Entry	R	X	Time (min)	Yield (%)^b^	M.p. (°C)		TOF (min^−1^)^c^
					Found	Reported	
1	H	I	5	98	68–70	68–70^58^	18.490
2	4-OCH_3_	I	8	95	87–89	88–90^58^	11.202
3	4-CH_3_	I	6	97	45–47	44–46^58^	15.251
4	2-CO_2_H	I	11	97	107–109	107–109^59^	8.319
5	H	Br	7	96	68–70	68–70^58^	12.938
6	4-CH_3_	Br	9	98	45–47	44–46^58^	10.272
7	4-OH	Br	10	98	161–163	163–164^60^	9.245
8	4-CHO	Br	12	97	55–57	54–56^61^	7.265
9	4-CN	Br	13	97	84–86	85–86^60^	7.039
10	4-CO_2_H	Br	13	94	226–228	224–228^62^	6.821
11	4-NO_2_	Br	9	97	111–113	112–114^58^	10.167
12	4-NH_2_	Br	10	97	51–53	50–53^61^	9.150
13	4-SH	Br	11	95	109–111	110–111^63^	8.147
14	3-CHO	Br	10	94	52–54	53–54^64^	8.867
15	4-Cl	Br	10	95	71–73	71–73^65^	8.962
16	H	Cl	15	94	162–164	163–164^60^	5.911

^a^Reaction conditions: aryl halide (1 mmol), PhB(OH)_2_ (1 mmol), base (3 mmol), Pd-BisPyP@bilayer-SiO_2_@NMP (5 mg, 1.06 mol%), PEG-400 (2 mL).

^b^Isolated yield.

^c^Turnover frequency.

For the Stille reaction, the Pd-BisPyP@bilayer-SiO_2_@NMP catalyst also displays a high activity in PEG-400 solvent through 0.5 mmol of triphenyltin chloride (Ph_3_SnCl). As the data in Table 6 reflect, the results are similar to those related to the Suzuki reaction.

**Table 6:** Preparation of biphenyl derivative from Stille reaction using Pd-BisPyP@bilayer-SiO_2_@NMP^a^.


Entry	R	X	Time (min)	Yield (%)^b^	M.p. (°C)		TOF (min^−1^)c
					Found	Reported	
1	H	I	7	98	68–70	68–70^58^	13.207
2	4-OCH_3_	I	9	92	87–89	88–90^58^	9.643
3	4-CH_3_	I	10	97	45–47	44–46^58^	9.150
4	H	Br	8	98	68–70	68–70^58^	11.556
5	4-CH_3_	Br	14	97	45–47	44–46^58^	6.536
6	4-OH	Br	10	96	161–163	163–164^60^	9.056
7	4-CHO	Br	18	93	55–57	54–56^61^	4.874
8	4-CN	Br	11	98	84–86	85–86^60^	8.404
9	H	Cl	20	90	162–164	163–164^60^	4.245
10	4-Cl	Br	12	90	71–73	71–73^65^	7.075

^a^Reaction conditions: aryl halide (1 mmol), Ph_3_SnCl (0.5 mmol), base (3 mmol), Pd-BisPyP@bilayer-SiO_2_@NMP (5 mg, 1.06 mol%), PEG-400 (2 mL).

^b^Isolated yield.

^c^Turnover frequency.

**Scheme 2:**
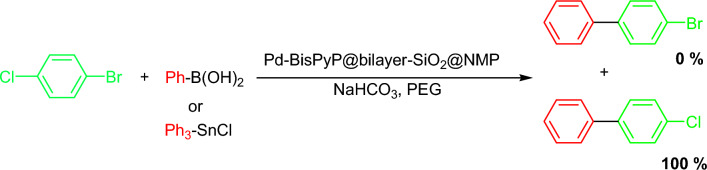
Selective coupling of 1-bromo-4-chorobenzene with phenylboronic acid in the presence of Pd-BisPyP@bilayer-SiO_2_@NMP.

To demonstrate the selectivity of our catalyst in both Suzuki and Stille coupling methods, 1-bromo-4-chorobenzene was coupled with PhB(OH)_2_ and Ph_3_SnCl (Table 5 Entry 15 and Table 6 Entry 10, respectively). The results showed that the coupling occurs only with the bromo functional group, meaning the chloro functional group remains intact during both cross-coupling reactions (Scheme 2).

**Scheme 3:**
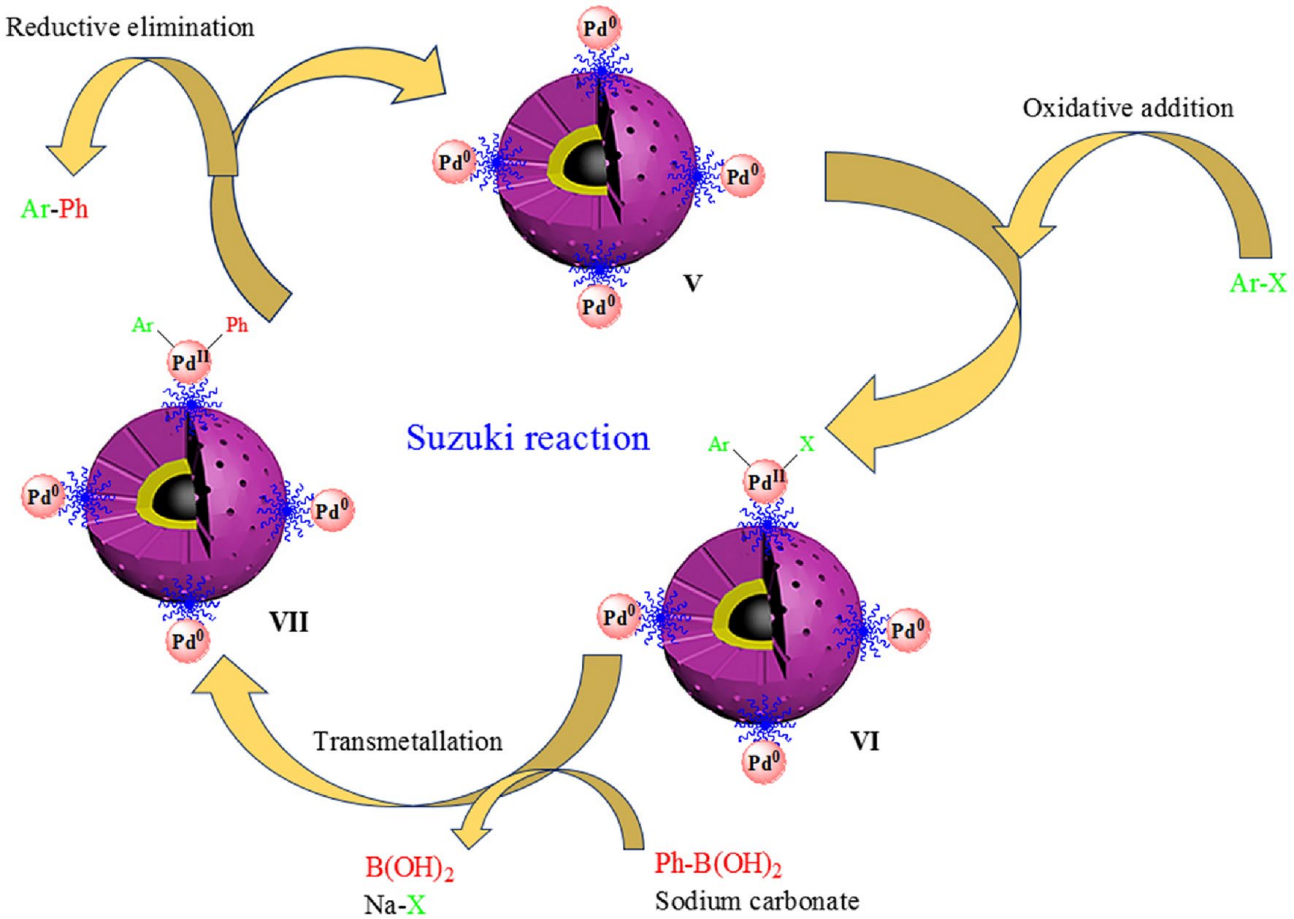
The suggested catalytic cycle of Suzuki reaction in the presence of Pd-BisPyP@bilayer-SiO_2_@NMP.

Scheme 3 shows the catalytic cycle of the Suzuki reaction in the presence of Pd-BisPyP@bilayer-SiO_2_@NMP catalyst in the following 3 steps, which is supported by the literature^65^: (1) the oxidative addition of Pd to Ar-X (aryl halide), and formation of organopalladium **VI** in the vicinity of Na_2_CO_3_ (2) the transmetallation of **VI** gives intermediate **VII** in the presence of phenylboronic acid, and finally (3) product formation and regeneration of palladium catalyst (**V**) by reductive elimination of intermediate **VII**.

### Reusability of catalyst

**Figure 12:**
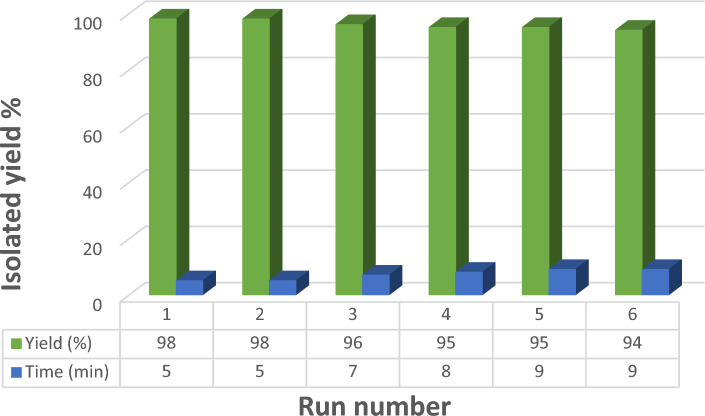
The recycling experiment of Pd-BisPyP@bilayer-SiO_2_@NMP in the coupling of iodobenzene with phenylboronic acid (Suzuki reaction).

It is critical to recover and reuse catalysts in green catalytic processes for economic and environmental reasons. As a consequence, the reusability of Pd-BisPyP@bilayer-SiO_2_@NMP was investigated in the coupling of iodobenzene with phenylboronic acid, and the findings are shown in Fig. 12. After the completion of the reaction, the catalyst was separated using an external magnet and washed with ethyl acetate to extract the product for this investigation. The recovered catalyst was reused up to six times without losing substantial catalytic activity. The average isolated yield for six consecutive cycles is 96%, indicating that this catalyst is recyclable.

### Catalyst leaching study

ICP-OES and hot filtration tests were used to investigate palladium leaching from Pd-BisPyP@bilayer-SiO_2_@NMP. The quantity of palladium in the fresh and reused catalysts was 2.123 × 10^–3^ mol g^−1^ and 2.109 × 10^–3^ mol g^−1^, respectively, according to ICP-OES analysis, indicating that palladium leaching of this catalyst is insignificant. A hot filtration test for the coupling of iodobenzene with phenylboronic acid was explored to assess the stability and heterogeneous nature of Pd-BisPyP@bilayer-SiO_2_@NMP under the reaction circumstances. In this investigation, 67% of the product was produced in half the reaction duration (the reaction time is 5 min). The reaction was then repeated, and at halftime, the catalyst was separated, and the filtrated solution was allowed to complete the reaction without a catalyst for another 2.5 min. After that, only 69% of biphenyl was recovered as a product. These trials show that palladium leaching did not occur^66^.

### Comparison of the catalyst

The efficiency of Pd-BisPyP@bilayer-SiO_2_@NMP as a catalyst was demonstrated by comparing it with the catalysts reported in the literature. Results for the coupling of iodobenzene with phenylboronic acid in the presence of Pd-BisPyP@bilayer-SiO_2_@NMP and previously reported catalysts are summarized in Table 7. In this comparison, various parameters such as reaction condition, reaction times and yields were compared. As shown in Table 7, Pd-BisPyP@bilayer-SiO_2_@NMP showed high effectivity than other catalysts in terms of reaction time and isolated yield.

**Table 7:** Comparison of the activity of Pd-BisPyP@bilayer-SiO_2_@NMP in the reaction of iodobenzene and phenylboronic acid with other catalysts.

Catalyst	Conditions	Time (min)	Yield (%)	References
Mag-IL-Pd	K_2_CO_3_, H_2_O, 60 °C	360	95	^67^
Guanidine/Pd(OAc)_2_	K_2_CO_3_, H_2_O EtOH, rt	1200	99	^68^
NAS@Cu	CS_2_CO_3_, EtOH, 80 °C	120	97	^39^
Pd(II)-NHC complex	Cs_2_CO_3_, DMF, 100 °C	1440	99	^69^
NHC-Pd(II) complex	Cs_2_CO_3_, THF, 80 °C	720	88	^70^
Pd/Au NPs	K_2_CO_3_, EtOH/H_2_O, 80 °C	1440	88	^71^
CA/Pd(0)	K_2_CO_3_, H_2_O, 100 °C	120	94	^72^
Pd@SBA-15/ILDABCO	K_2_CO_3_, H_2_O, 80 °C	90	97	^73^
BisPyP@ySiO_2_@xSiO_2_@NMP	Na_2_CO_3_, PEG, 70 °C	5	99	[This work]

## Conclusions

In this research, we have presented a new organic–metallic nanocatalyst (Pd-BisPyP@bilayer-SiO_2_@NMP) with three unique characteristics, i.e., excellent performance in accelerating the reaction time, magnetic nature, and high porosity. These three properties make this magnetic mesoporous material a versatile and effectual catalyst. The catalytic performance of this catalyst was investigated in Suzuki and Stille cross-coupling reactions. These reactions were carried out in eco-friendly conditions and decorated with the following benefits: high purity of products without generation of side products, simple separation of products and catalyst from each other, the phosphine-free structure of the catalyst, the possibility of recovering and reusing the catalyst up to 6 times, high efficiency under relatively moderate conditions, the use of PEG as a green solvent, and not leaching the catalyst, especially the metal parts (Pd), with the confirmation of hot filtration and ICP-OES methods.

## Data Availability

The data that support the findings of this study are available on request from the corresponding author.
